# Improved Cardiovascular and Cardiometabolic Risk in Patients With Type 1 Diabetes and Autoimmune Polyglandular Syndrome Switched From Glargine to Degludec Due to Hypoglycaemic Variability

**DOI:** 10.3389/fendo.2018.00428

**Published:** 2018-07-26

**Authors:** Valentina Guarnotta, Giulia Di Bella, Giuseppe Pillitteri, Alessandro Ciresi, Carla Giordano

**Affiliations:** Biomedical Department of Internal and Specialist Medicine, Section of Diabetes, Endocrinology and Metabolism, University of Palermo, Palermo, Italy

**Keywords:** type 1 diabetes, insulin therapy, hypoglycaemia, cardiovascular risk, glargine, degludec

## Abstract

**Background:** Cardiovascular disease is a frequent complication of type 1 diabetes (T1D). We evaluated the effectiveness of switching from glargine to degludec in reducing the cardiovascular risk factors, the Framingham risk score (FRS) and visceral adiposity index (VAI) in patients with T1D and autoimmune polyglandular syndrome (APS).

**Methods:** We selected 66 T1D outpatients who had been on stable treatment with glargine for at least 5 years. Among them, 30 patients maintained glargine (group A), while 36 were switched to degludec (group B) for 12 months. At baseline and after 12 months of observation, clinical and metabolic parameters, insulin dose, 30-days blood glucose (BG) self monitoring, VAI and FRS were obtained.

**Results:** At baseline, patients in group B had more hypoglycaemic episodes and prevalence of hypertension than those in group A. After 12 months on degludec, patients in group B had a significant decrease in BMI (*p* = 0.003), waist circumference (*p* < 0.001), total daily insulin as U/day and U/kg (*p* = 0.001 for both), basal insulin as U/day and U/kg (*p* = 0.001 for both), HbA1c (*p* < 0.001), mean (*p* = 0.035) and standard deviation of daily BG (*p* = 0.017), mean pre-meal BG (*p* = 0.016), number of hypoglycaemic episodes (*p* = 0.001), VAI (*p* = 0.012) and FRS (*p* = 0.019) and a significant increase in HDL-C (*p* < 0.001), compared to baseline. At 12 months of treatment a significant decrease in BMI (*p* = 0.017), WC (*p* = 0.003), SBP (*p* = 0.001), DBP (*p* = 0.005), basal insulin as U/day (*p* = 0.018) and U/kg (*p* = 0.045), HbA1c (*p* = 0.040) and FRS (*p* = 0.010) was observed in group B compared to group A.

**Conclusions:** Our preliminary data suggest that 12 months' treatment with degludec is associated with an improvement of glycaemic control, cardiometabolic and cardiovascular risk, compared to glargine, in patients with T1D and APS.

## Introduction

Increased cardiovascular risk is widely recognized as a complication of type 1 diabetes (T1D) due to multiple risk factors, such as HbA1c, blood pressure, lipids, smoking, and visceral obesity (1). Among all of them, glycaemic control appears to be strictly involved in the development of micro and macrovascular disease in T1D, with great impact on mortality (2, 3).

T1D is a complex disease characterized by the presence of many factors initiating or modulating immune response leading to development of the disease (4). It can be associated with other autoimmune diseases in the spectrum of the autoimmune polyglandular syndrome (APS), which is characterized by poor quality of life and increased morbidity and mortality, compared to T1D alone (5). The most frequent comorbidities of T1D in the APS spectrum include Hashimoto's thyroiditis and Graves' disease collectively referred to as autoimmune thyroid diseases (15–30%), celiac disease (4–9%), autoimmune gastritis/pernicious anemia (5–10%), Addison's disease (0.5%), and vitiligo (2–10%) (6).

The management of T1D is based on insulin analogs which should avoid excessive glycaemic variability ensuring flat pharmacological action and longer duration of action (7, 8). Insulin degludec is a new generation ultra long-acting insulin characterized by self-association into stable di-hexamer complexes (9). As a result, degludec has no peak activity and a duration of action longer than 24 h and can be used as once-daily insulin (10). The efficacy of insulin degludec for treatment of T1D and its non-inferiority to glargine has been demonstrated in several clinical trials (11–13). Degludec treatment has been associated with a decrease in HbA1c (14), in daily insulin dose and nocturnal hypoglycaemia, notably in the maintenance period (15, 16), with an adverse profile similar to glargine (17), also in terms of cardiovascular events (18).

The superiority of insulin degludec vs. glargine in terms of hypoglycaemic risk has been widely demonstrated in patients both with T1D (SWITCH 1) (19) and with T2D (SWITCH 2) (18, 20). Interestingly, an association between fasting glucose variability, higher risk of hypoglycaemia and total mortality has also been reported (21).

However, the effects of degludec on weight and visceral fat adiposity have been poorly investigated. The visceral adiposity index (VAI), a valuable indicator of visceral adiposity function and insulin sensitivity, has been demonstrated to be an independent predictor of cardiovascular disease and an easy tool to show a condition of cardiometabolic risk (22).

The current study aimed to evaluate the effectiveness of switching from glargine to degludec in a real-world setting, in reducing a composite index of cardiovascular risk [the Framingham risk score (FRS)] (23) and of cardiometabolic risk, the VAI, in patients with T1D and APS for a 12-month period.

## Materials and methods

### Study participants

We retrospectively extracted data of 66 T1D and APS outpatients who were on glargine basal treatment, consecutively referred to the Division of Endocrinology of Palermo University from January 2015 to December 2017. Specifically, we carefully selected data of 30 patients who were on glargine treatment and maintained this basal insulin (group A) and 36 patients who were on glargine treatment and were switched to degludec (group B), for a 12 month-period.

Patients were matched for gender (*p* = 0.512), age (*p* = 0.850), BMI (p=0.103), and waist circumference (WC) (*p* = 0.849). All patients had a disease duration of at least five years. Inclusion criteria were the following: age 25–65 years; diagnosis of T1D; ongoing daily glargine, in addition to mealtime insulin, from at least 5 years; HbA1c >7.0% and/or frequent hypoglycaemic episodes. Exclusion criteria were as follows: hypoglycaemia unawareness, acute illnesses or infections, inability to provide informed consent, pregnancy and lactation. The switch to degludec was judged to be appropriate on clinical grounds in those patients on glargine treatment who had wide glycaemic variability and high tendency to have hypoglycaemic episodes. Diagnosis of T1D was done according to the ADA guidelines (24) and any associated autoimmune disease was screened based on clinical symptoms and serological testing (25). Hypoglycaemia was defined as a measured blood glucose (BG) of ≤70 mg/dl (3.9 mmol/L) symptomatic or not, according to the ADA criteria (24).

Overall, all patients had APS-2. As recently reported, APS can be broadly categorized as rare monogenic forms, such as APS-1, and a more common polygenic variety, APS-2 (26). APS-1 is characterized by the development of at least two of three cardinal components during childhood chronic mucocutaneous candidiasis, hypoparathyroidism, and primary adrenal insufficiency (Addison's disease) and other associated manifestations. APS-2 is characterized by at least two of the following three endocrinopathies: type 1 diabetes, autoimmune thyroid disease, and Addison's disease. Celiac disease, alopecia, vitiligo, primary ovarian insufficiency, and pernicious anemia also are commonly observed in this syndrome.

In group A, one patient had Addison's disease, 20 had autoimmune thyroiditis, 14 had hypothyroidism, 7 had celiac disease, 3 had vitiligo, 2 had autoimmune atrophic gastritis. In group B, 5 patients had Addison's disease, 26 had autoimmune thyroiditis, 10 with hypothyroidism, 9 had celiac disease, 3 had vitiligo, and 5 had autoimmune atrophic gastritis.

Patients with hypothyroidism were on treatment with levo-thyroxine at the average dose of 1 mcg/kg; patients with adrenal insufficiency were on treatment with cortisone acetate or hydrocortisone treatment, at the mean dose of 30 and 25 mg respectively, administered twice or three times a day. In addition, patients with adrenal insufficiency were on stable treatment with fludrocortisone (0.05–0.1 mg/day, once).

### Patients with celiac disease were on a stable gluten-free diet

During the 12 months of observation, the dose of levothyroxine and glucocorticoids was not changed and patients maintained good control of all the T1D autoimmune-associated diseases.

Primary objective of the current study was to evaluate the effectiveness of degludec treatment in reducing the cardiovascular and cardiometabolic risk compared with glargine. Secondary obejectives were to evaluate: (1) changes in HbA1c from baseline to 12 months of follow-up; (2) change in clinical parameters; (3) changes in basal insulin requirement and risk of hypoglycaemic episodes.

All procedures were in accordance with the ethical standards of the responsible committee on human experimentation (institutional and national) and with the Helsinki Declaration of 1964, as revised in 2013. Approval was obtained from the Ethics Committee of the University of Palermo. At the time of hospitalization, an informed consent for the scientific use of the data was obtained from all patients for being included in the study.

### Study design

Before switching to degludec, self-monitored blood glucose (SMBG) profiles of the preceding 30 days were extracted from our database. SMBG profiles were recorded by glucometer download in 23/66 patients and from diaries in 43/66 patients.

At switch time, degludec was started with a dose reduction of 20% compared to the glargine dose, followed by individual dosage adjustment based on the glycaemic response communicated weekly by telephone or email. Individualized fasting glucose targets were between 90 and 140 mg/dl and were not modified after switching to degludec. Insulin degludec was administered at bedtime. In the same way, individual dosage adjustment based on glycaemic values was carried out in patients maintaining glargine.

BG levels of at least twice/day were extracted from our database, before and 2 h after breakfast, lunch, and dinner, considering the last month of SMBG values. In a subgroup of 10 patients the nocturnal BG, measured between midnight and 6 am in the morning, was available.

For each group, the following indexes were calculated: mean and standard deviation (SD) of daily BG, mean fasting, pre and post-meal glucose values, hypoglycaemic episodes.

Anthropometric parameters such as BMI, systolic (SBP) and diastolic blood pressure (DBP) and WC, measured at the midpoint between the lower rib and the iliac crest, were extracted. In addition, lipids [total cholesterol (TC), HDL cholesterol (HDL-C), LDL cholesterol (LDL-C), and triglycerides (TG)], HbA1c and fasting glycaemia were obtained. VAI was calculated according to gender, where TG levels were expressed in mmol/l and HDL levels were expressed in mmol/l:

- Males VAI = [WC/39.68 + (1.88 × BMI)] × (TG/1.03) × (1.31/HDL);
- Females VAI = [WC/36.58 + (1.89 × BMI)] × (TG/0.81) × (1.52/HDL) (22).

The FRS for estimating the 10-year risk for cardiovascular events was calculated on the basis of age, gender, total, and HDL cholesterol, blood pressure and smoking status (23).

In addition the percentage of patients with arterial hypertension, defined as use of antihypertensive medication and/or presence of SBP ≥ 130 mm Hg or DBP ≥ 85 mm Hg, visceral obesity, defined as WC >102 cm in men and >88 cm in women, hypercholesterolemia, defined as LDL-cholesterol >3.36 mmol/L and low HDL-cholesterol, defined as a value <1.04 mmol/L in men and <1.30 mmol/L in women (27) was collected in both groups.

The retrospective analysis covered 12 months of observation. At the end, the same parameters measured at baseline, together with the SMBG profile of the last 30 days, were extracted.

The differences in clinical and metabolic parameters between groups at baseline and after 12 months of follow-up, as well as the difference from baseline to 12 months on each group, were evaluated.

The changes in parameters from baseline to 12 months were calculated and expressed as Δ values.

No adverse drug reactions were recorded.

### Assays

Glycaemia, HbA1c and lipids were measured by standard methods (Modular P800, Roche, Milan). LDL-C levels were measured using the Friedewald formula [TC – (HDL + (TG/5)].

The conversion factors for the International System (SI) were as follows: TC and HDL-C mg/dl vs. mmol/l: 0.0259; TG mg/dl vs. mmol/l: 0.0113; HbA1c % vs. mmol/mol: 10.93 %−23.5.

### Statistical analysis

The SPSS version 19 (SPSS, Inc.) was used for data analysis. Data were presented as mean ± SD or rates and proportions. The normality of distribution of the quantitative variables was assessed using the Kolmogorov-Smirnov test. The differences between the two groups at baseline and after 12 months were evaluated with *t*-Student for quantitative variables and χ^2^ for trend for categorical variables.

The differences between paired continuous variables from baseline to 12 months in each group were evaluated using the Student's *t*-test.

Comparative statistical evaluations between the two independent variables time of observation (baseline-12 months) and treatment (degludec/glargine) were accomplished with two-way ANOVA. The main effect of each factor was tested as well as the interaction within both factors. Univariate correlations among continuous variables with normal distribution were determined by Pearson's test. A *p* < 0.05 was considered statistically significant.

## Results

At baseline, no differences between groups A and B were observed with regard to diabetes complications, type of APS, smoking, central obesity, hypercholesterolemia, low HDL, duration of diabetes, total daily, basal and mealtime insulin doses and HbA1c (Table 1). Patients in group B had more prevalence of hypertension (*p* = 0.034) and hypoglycaemic events (*p* < 0.001) than group A (Table 1).

**Table 1:** General characteristics of the two groups of patients at baseline.

	**All baseline (*N* = 66)**	**Group A baseline (*N* = 30)**	**Group B baseline (*N* = 36)**	p^*^
	**Subjects (%)**	**Subjects (%)**	**Subjects (%)**	
Gender				
Male	18 (27.3%)	23 (76.6%)	25 (69.4%)	0.512
Female	48 (72.7%)	7 (23.3%)	11 (30.6%)	
Complications				
Retinopathy	13 (19.7%)	3 (10%)	10 (27.8%)	0.071
Nephropathy	11 (16.7%)	5 (16.7%)	6 (16.7%)	0.627
Neuropathy	7 (10.6%)	2 (6.7%)	5 (13.9%)	0.296
Smoking	13 (19.7%)	5 (16.7%)	8 (22.2%)	0.572
Arterial Hypertension	5 (7.6%)	0	5 (13.9%)	0.034
Visceral obesity	16 (24.2%)	8 (26.7%)	8 (22.2%)	0.675
Hypercholesterolemia	16 (24.2%)	8 (26.7%)	8 (22.2%)	0.722
Low HDL-cholesterol	18 (27.3%)	10 (33.3%)	8 (22.2%)	0.226
	***Mean** ± **SD***	***Mean** ± **SD***	***Mean** ± **SD***	
Age (years)	41.7 ± 13.8	44.9 ± 13.9	39 ± 13.4	0.850
Duration of disease (years)	14.4 ± 9.26	11.6 ± 9.3	15.8 ± 9.01	0.130
Total daily insulin (UI/day)	45.5 ± 15.8	45.9 ± 18.8	40.02 ± 12.8	0.448
Total daily insulin (UI/kg)	0.70 ± 0.21	0.67 ± 0.29	0.72 ± 0.16	0.656
Basal insulin (UI/day)	19.1 ± 7.9	19.5 ± 9.2	15.6 ± 5.8	0.155
Basal insulin (UI/kg)	0.29 ± 0.11	0.29 ± 0.1	0.29 ± 0.09	0.943
Total prandial insulin (UI/day)	26.4 ± 10.6	22.1 ± 17.7	24.3 ± 10.1	0.107
Total prandial insulin (UI/kg)	0.41 ± 0.14	0.40 ± 0.15	0.42 ± 0.13	0.575
BMI (kg/m^2^)	24.3 ± 4.24	25.3 ± 4.18	23.6 ± 4.18	0.103
Waist circumference (cm)	86.9 ± 9.96	86.7 ± 11.3	87.2 ± 8.8	0.849
HbA1c (mmol/mol)	75.8 ± 20.2	74.5 ± 22.2	77.3 ± 18.7	0.517
Standard deviation of daily blood glucose (SD)	49.2 ± 12.4	42.1 ± 4.64	58.4 ± 14.1	0.010
N blood glucose (BG) ≤ 70 mg/dl (24h/day)	1.97 ± 1.90	0.78 ± 1.08	2.65 ± 1.93	<0.001

* *p, difference between patients in group A and B at baseline*.

During the 12 months of observation, patients in group A had an increase in WC (*p* = 0.001), without any other significant differences (Table 2). By contrast, patients of group B had a significant decrease in BMI (*p* = 0.003), WC (*p* < 0.001), total daily insulin as U/day and U/kg (*p* = 0.001 for both), basal insulin as U/day and U/kg (*p* = 0.001 for both) and a significant increase in HDL-C (*p* < 0.001) (Table 2). With regard to glucose control, a decrease in HbA1c (*p* < 0.001), mean (*p* = 0.035) and SD (*p* = 0.017) of daily BG, mean pre-meal BG (*p* = 0.016) and number of hypoglycaemia episodes (*p* = 0.001) was observed in group B (Table 2).

**Table 2:** Clinical and metabolic parameters, insulin requirement and glucose control in group A and B at baseline and after 12 months of treatment.

	**Group A (*****N*** = **30)**			**Group B (*****N*** = **36)**			
	**Baseline Mean ± SD**	**12 months Mean ± SD**	p^*^	**Baseline Mean ± SD**	**12 months Mean ± SD**	p^**^	p^***^
**CLINICAL PARAMETERS**							
BMI (Kg/m^2^)	25.3 ± 4.18	25.6 ± 3.77	0.282	23.6 ± 4.18	22.9 ± 4.17	0.040	0.017
Waist circumference (cm)	86.7 ± 11.3	90.5 ± 9.31	0.001	87.2 ± 8.8	83.7 ± 8.5	<0.001	0.003
Systolic blood pressure (mmHg)	115.1 ± 14.6	118.5 ± 11.4	0.180	110.9 ± 11.6	109.4 ± 10.5	0.412	0.001
Diastolic blood pressure (mmHg)	69.1 ± 8.41	72.6 ± 9.07	0.118	63.6 ± 8.07	66.5 ± 8.09	0.070	0.005
**METABOLIC PARAMETERS**							
Total cholesterol (mmol/l)	4.95 ± 0.98	5 ± 0.95	0.760	4.82 ± 0.88	4.61 ± 0.77	0.081	0.069
HDL cholesterol (mmol/l)	1.50 ± 0.4	1.60 ± 0.45	0.243	1.58 ± 0.49	1.68 ± 0.51	0.001	0.790
Triglycerides (mmol/l)	1.19 ± 0.72	1.10 ± 0.71	0.492	0.98 ± 0.43	0.88 ± 0.38	0.095	0.109
LDL cholesterol (mmol/l)	2.9 ± 0.96	2.87 ± 0.98	0.863	2.79 ± 0.81	2.66 ± 0.73	0.285	0.319
**INSULIN REQUIREMENT**							
Total daily insulin (UI/day)	45.9 ± 18.8	48.03 ± 21.5	0.105	45.2 ± 12.9	40.02 ± 12.8	0.001	0.066
Total daily insulin (UI/kg)	0.67 ± 0.29	0.72 ± 0.30	0.058	0.72 ± 0.16	0.65 ± 0.17	0.001	0.166
Basal insulin (UI/day)	19.5 ± 9.1	20.01 ± 8.8	0.051	18.7 ± 6.8	15.6 ± 5.8	<0.001	0.018
Basal insulin (UI/kg)	0.29 ± 0.11	0.30 ± 0.10	0.275	0.29 ± 0.09	0.25 ± 0.09	<0.001	0.045
Total prandial insulin (UI/day)	26.3 ± 11.3	28 ± 14.3	0.217	26.5 ± 10.1	24.3 ± 10.1	0.053	0.232
Total prandial insulin (UI/kg)	0.40 ± 0.15	0.42 ± 0.21	0.186	0.42 ± 0.13	0.39 ± 0.14	0.073	0.394
**GLUCOSE CONTROL**							
HbA1c (mmol/mol)	74.5 ± 22.2	73.5 ± 20.5	0.707	77.3 ± 18.7	64.8 ± 12.2	<0.001	0.040
N. of blood glucose (BG)/day (total)	2.83 ± 1.11	2.63 ± 0.96	0.517	2.69 ± 1.03	2.55 ± 0.73	0.523	0.711
Mean daily BG (mg/dl)	145.1 ± 32.6	151.8 ± 34.7	0.488	168.9 ± 64	140.1 ± 30.1	0.035	0.608
SD (mg/dl)	42.1 ± 4.64	45.6 ± 6.26	0.399	58.4 ± 14.1	41.1 ± 11.7	0.017	0.147
Mean fasting BG (mg/dl)	163.3 ± 54.1	174.9 ± 70.3	0.494	172 ± 62.9	157.4 ± 41.3	0.270	0.245
Mean pre-meal BG (mg/dl)	171.9 ± 123.3	152.2 ± 33.9	0.519	188.8 ± 50.6	155.1 ± 34.3	0.016	0.793
Mean post-meal BG (mg/dl)	141.3 ± 39.5	145.8 ± 40.4	0.656	145.9 ± 57.8	136.2 ± 42.8	0.365	0.360
N. of BG ≤ 70 mg/dl (24h/day)	0.78 ± 1.08	0.63 ± 0.76	0.506	2.65 ± 1.93	0.82 ± 0.58	0.001	0.310

* *p, difference between baseline and 12 months of treatment in group A*.

** *p, difference between baseline and 12 months of treatment in group B*.

*** *p, difference between groups A and B after 12 months of treatment*.

**Figure 1:**
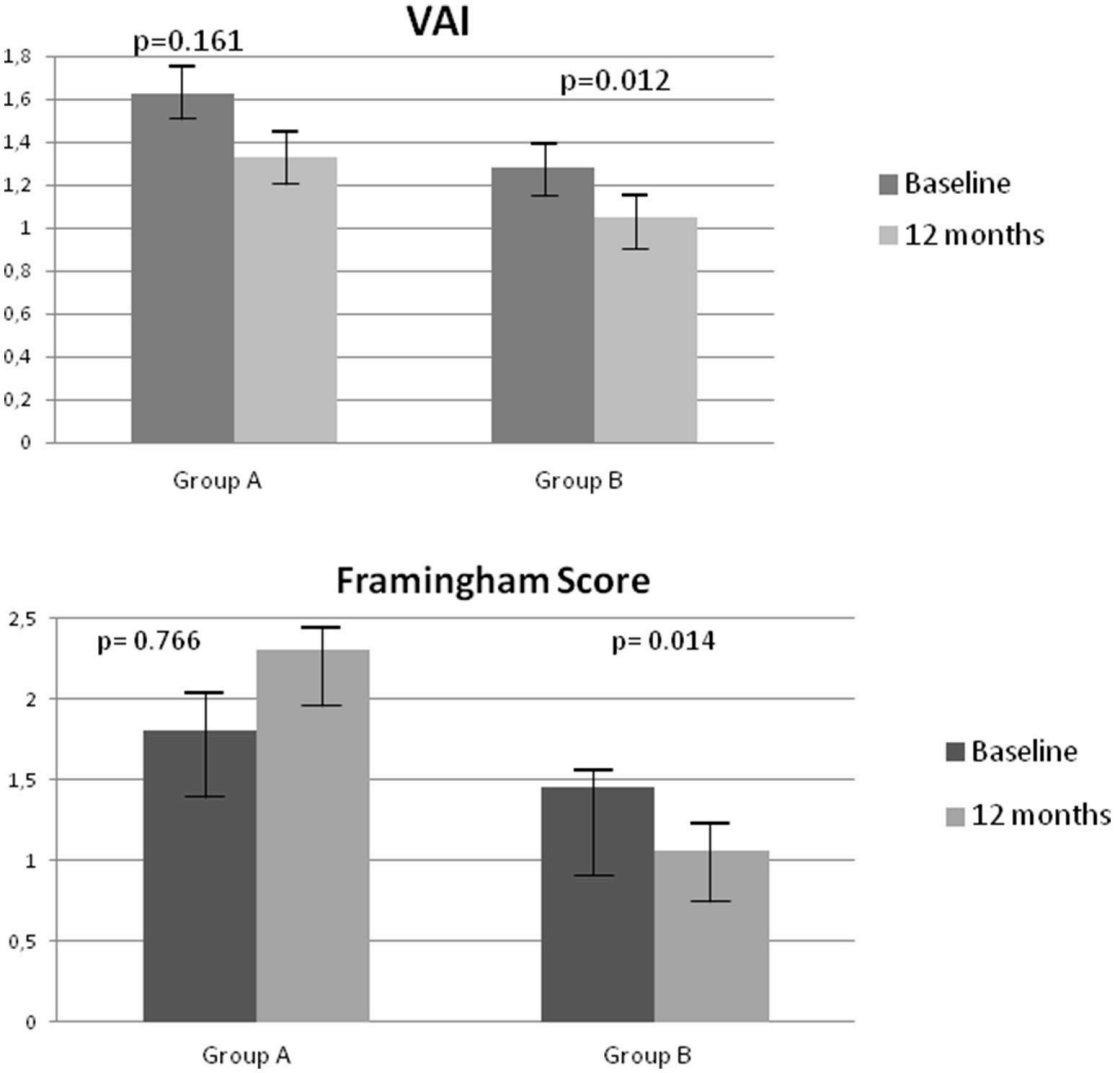
Changes in Visceral Adiposity Index (VAI) and Framingham Risk Score (FRS) at baseline and after 12 months in groups A and B.

In addition, a significant decrease in VAI (*p* = 0.012) and FRS (*p* = 0.019) was observed after 12 months on degludec in group B (Figure 1).

**Figure 2:**
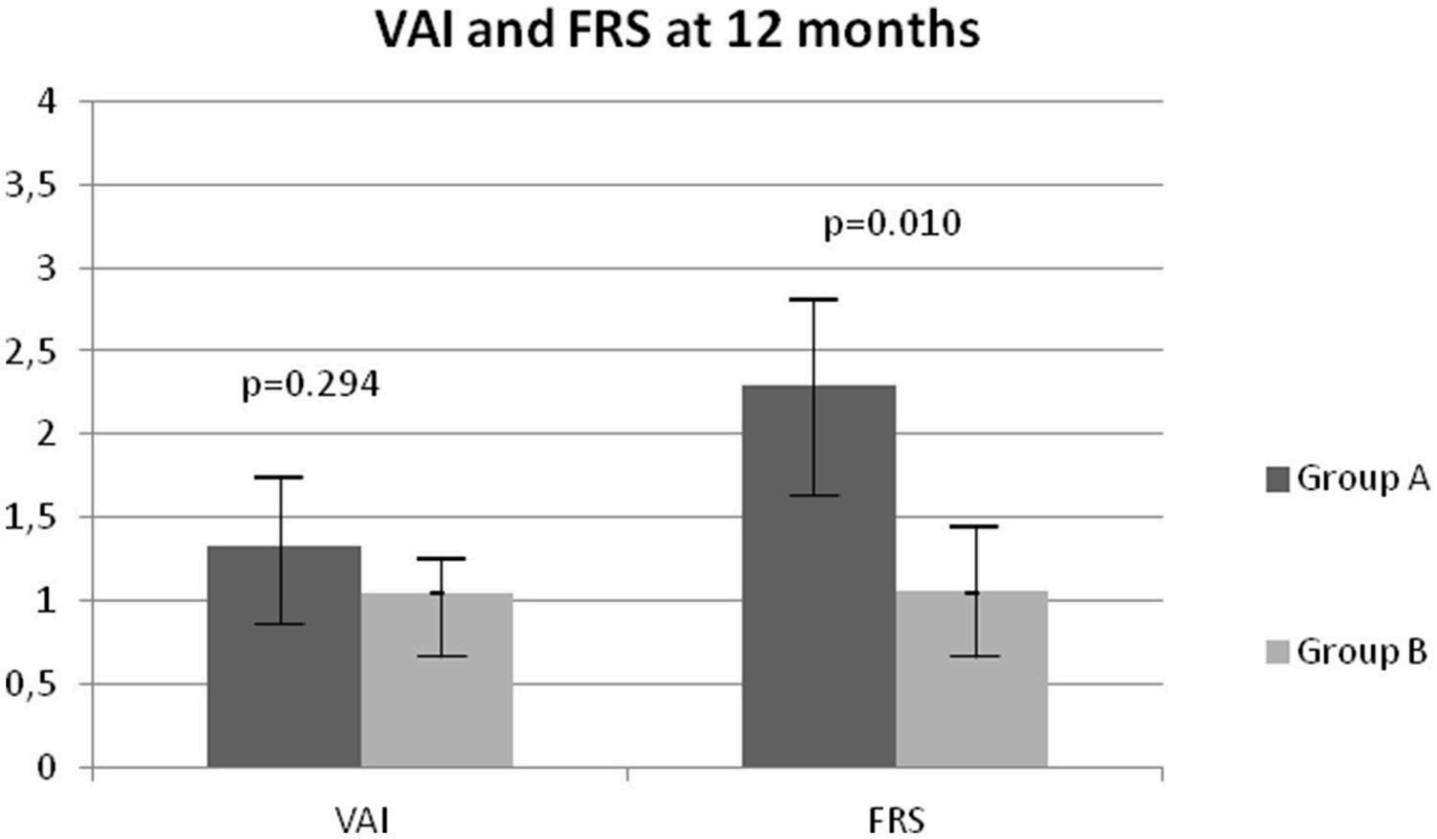
Comparison of Visceral Adiposity Index (VAI) and Framingham Risk Score (FRS) after 12 months in groups A and B.

At 12 months of treatment a significant decrease in BMI (*p* = 0.017), WC (*p* = 0.003), SBP (*p* = 0.001), DBP (*p* = 0.005), basal insulin as U/day (*p* = 0.018) and U/kg (*p* = 0.045), HbA1c (*p* = 0.040) and FRS (*p* = 0.010) was observed in group B compared to group A (Table 2 and Figure 2).

Two-way ANOVA analysis revealed there was a significant effect of treatment over time of observation, on the following parameters: BMI (*p* = 0.035), WC (*p* ≤ 0.001), HDL-cholesterol (*p* ≤ 0.001), total insulin as U/day (*p* < 0.001), total insulin as U/kg (*p* = 0.041), basal insulin as U/day (*p* ≤ 0.001), basal insulin as U/kg (*p* = 0.048), HbA1c (*p* = 0.015), number of BG ≤ 70 mg/dl (24 h/day) (*p* = 0.016), VAI (*p* < 0.001), FRS (*p* < 0.001). No significant interactions between treatment and time of observation were found (results not shown).

After 12 months of degludec treatment, Δ_weight showed a significant positive correlation with Δ_daily insulin doses (*r* = 0.282, *p* = 0.022) and with Δ_basal insulin doses (*r* = 0.380, *p* = 0.002), Δ_BMI and Δ_WC showed a positive correlation with Δ_basal insulin doses (*r* = 0.588, *p* = 0.034 and *r* = 0.416, *p* = 0.022, respectively), while no correlations were found among Δ _BMI and Δ_WC with Δ_daily insulin doses (Table 3).

**Table 3:** Bivariate correlations between Δ _weight, Δ _BMI and Δ _WC with Δ _daily insulin doses (U/day) and Δ _basal insulin doses (U/day) in patients of group B.

	Δ**_daily insulin doses**		Δ **_basal insulin doses**	
	***r***	***p***	***r***	***p***
**CLINICAL PARAMETERS**				
Δ_weight	0.282	0.022	0.380	0.002
Δ_BMI	0.234	0.422	0.588	0.034
Δ_WC	0.052	0.765	0.416	0.022

## Discussion

In this retrospective study we found that switching from glargine to degludec for 12 months in patients with T1D and APS is associated with a decrease in BMI and WC, HbA1c, hypoglycaemic episodes, insulin requirement, improvement of glucose variability and increase in HDL-C, but most of all with an improvement of VAI and FRS.

The decrease of BMI and WC is an interesting finding. A systematic meta-analysis of randomized controlled trials comparing degludec and glargine, both in patients with T1D and T2D, did not show any change in BMI and WC (16). However, it is well-known that glargine 100 U/ml treatment is associated with weight gain (28, 29), compared to other basal insulin such as detemir and glargine 300 U/ml (30, 31). The weight decrease in the group of patients switched to degludec seems to be related to the decrease of daily insulin and basal doses as demonstrated by the positive correlation of Δ_weight and Δ_daily and basal insulin. Indeed, it has been demonstrated that weight gain in patients with diabetes is related to many factors such as over-replacement of insulin, increased calorie intake, as an adaptive response to hypoglycaemia, and insulin resistance (29).

Degludec insulin has been reported to improve glucose control through a decrease in glycaemic variability and hypoglycaemic episodes and a decrease of insulin doses, notably in the maintenance period (14, 16, 32). In addition, a decrease in insulin requirement and an improvement of HbA1c has also been observed in patients switched from glargine to degludec, even though these results are still quite discordant (33, 34).

In addition, in the current study the comparison of 12 months of treatment of glargine vs. degludec showed that patients treated with degludec had a significant decrease in BMI, WC, HbA1c and total basal insulin, demonstrating the favorable effect of degludec on anthropometric parameters, but also glycometabolic control.

Interestingly, a significant decrease in VAI was also found. VAI has been demonstrated to be an accurate surrogate of cardiometabolic risk evaluation and to indirectly express altered production, release, and/or function of adipocytokines and inflammatory factors in patients with T2D and other endocrine disorders (22, 35–37). In addition, VAI has been demonstrated to predict insulin resistance (38) and may be a better surrogate of insulin sensitivity evaluation, in patients with T1D, than insulin requirement, which is influenced by residual pancreatic function and by variability in absorption of insulin in subcutaneous adipose tissue (39).

In our opinion, the significant decrease in VAI is related to the improvement of insulin sensitivity and decrease of insulin doses, which are indirectly correlated with the improvement of WC, BMI, and HDL-cholesterol.

With regard to cardiovascular risk, a decrease of FRS was observed in patients switched to degludec treatment. T1D is associated with an accelerated risk of cardiovascular disease compared to subjects without diabetes, mainly related to glycaemic control (40). Results from the Diabetes Control and Complications Trial (DCCT) showed that intensive management of hyperglycaemia is associated with slow progression of micro and macrovascular complications (2), even though despite the current use of intensive insulin treatment, the risk of cardiovascular disease (CVD) in patients with T1D still remains high, suggesting the importance of other factors, such as blood pressure, lipids, obesity and smoking (41). Based on this evidence, an assessment of risk factors for CVD and the use of a CVD risk-prediction algorithm could be useful. The FRS is the current recommended index to predict cardiovascular risk in the general population (24). However, there are some controversies about its use in people with diabetes for a possible underestimation of CVD risk. Indeed, the FRS does not include evaluation of glycaemic control and duration of diabetes. Studies evaluating the ability of predicting CVD risk score developed in a diabetic cohort and a score developed in the general population reported inconsistent results. In a study on 339 diabetic patients the FRS appeared to be more accurate than the UKPDS risk engine for predicting coronary heart disease, while in a British study on 428 patients with diabetes, the FRS underestimated the CVD risk compared to the UKPDS risk engine (42, 43). For these reasons, currently the most accurate parameter to estimate the CVD risk in patients with diabetes is quite uncertain, and in the absence of specific data to the contrary, an approach to evaluate the CVD risk in the diabetic population is to use the same CVD risk-assessment algorithm as for the general population (44). In addition, in our study we compared two populations of patients with T1D to observe the effects of two different basal insulin treatments on FRS modification. The improvement of FRS in patients switched from glargine to degludec is the result of the improvement of lipids, blood pressure, and visceral obesity, which are known cardiovascular risk factors, and that are indirectly influenced by the decrease of the insulin doses and the improvement of glycaemic control.

The main limitations of the present study are that it is not randomized and many selection bias may exist and the small number of patients enrolled. Patients were not blinded to the treatment and therefore their expectations on the new drug may have partially affected the results. The insulin titration was performed according to routine clinical practice by the caring diabetologist, without reference to a pre-specified algorithm. In addition, patients were not advised to change their dietary regimen and the SMBG device was not the same for all patients enrolled. Lastly, the cohort of patients is quite heterogeneous (patients with T1D and APS) and is characterized by different metabolic alterations, due to other hormonal deficiencies. However, all patients enrolled in the study were on stable replacement treatment and maintained good and stable hormonal control during the whole follow-up, thus not affecting, in our opinion, the outcomes of the study.

In conclusion, our preliminary data, extracted from real-life clinical practice, suggest that 12 months' treatment with degludec is associated with an improvement of glycaemic control, cardiometabolic and cardiovascular risk compared to glargine treatment in patients with T1D and APS. These patients represent a minority of patients characterized by high fragility and increased cardiovascular risk compared to those with T1D alone, thus benefitting more from switching to degludec insulin, notably in patients who had glycaemic variability and high hypoglycemic episodes. However, further case-controlled studies performed in a larger cohort of patients are required in order to verify our preliminary data.

## Ethics statement

This study was approved by the Institutional Review Board at the Faculty of Medicine of the University of Palermo and all participants signed the consent to use their data for scientific purpose.

## Author contributions

VG, GD, GP, AC, and CG had full control of the study design, data analysis and interpretation, and preparation of article. All authors were involved in planning the analysis and drafting the article. The final draft article was approved by all the authors.

### Conflict of interest statement

The authors declare that the research was conducted in the absence of any commercial or financial relationships that could be construed as a potential conflict of interest.
